# Relationships between Human Population Density and Burned Area at Continental and Global Scales

**DOI:** 10.1371/journal.pone.0081188

**Published:** 2013-12-16

**Authors:** Ioannis Bistinas, Duarte Oom, Ana C. L. Sá, Sandy P. Harrison, I. Colin Prentice, José M. C. Pereira

**Affiliations:** 1 Centro de Estudos Florestais, Instituto Superior de Agronomia, Universidade de Lisboa, Tapada da Ajuda, Lisboa, Portugal; 2 Department of Biological Sciences, Macquarie University, North Ryde, Australia; 3 Geography & Environmental Sciences, School of Human and Environmental Sciences, Reading University, Whiteknights, Reading, United Kingdom; 4 Grantham Institute for Climate Change, and Department of Life Sciences, Imperial College, Silwood Park Campus, Ascot, United Kingdom; University of Gävle, Sweden

## Abstract

We explore the large spatial variation in the relationship between population density and burned area, using continental-scale Geographically Weighted Regression (GWR) based on 13 years of satellite-derived burned area maps from the global fire emissions database (GFED) and the human population density from the gridded population of the world (GPW 2005). Significant relationships are observed over 51.5% of the global land area, and the area affected varies from continent to continent: population density has a significant impact on fire over most of Asia and Africa but is important in explaining fire over < 22% of Europe and Australia. Increasing population density is associated with both increased and decreased in fire. The nature of the relationship depends on land-use: increasing population density is associated with increased burned are in rangelands but with decreased burned area in croplands. Overall, the relationship between population density and burned area is non-monotonic: burned area initially increases with population density and then decreases when population density exceeds a threshold. These thresholds vary regionally. Our study contributes to improved understanding of how human activities relate to burned area, and should contribute to a better estimate of atmospheric emissions from biomass burning.

## Introduction

Fire is a natural process that has played a key role in the maintenance of natural ecosystems for millions of years, and regulates plant and animal population dynamics [1-3]. However, fire is also a tool used by people to transform the natural environment [4-6]. Humans are the dominant influence over most of the land surface today [7]. Prior to the industrial revolution only ca 5 % of the ice free land surface was used for agriculture and settlement. However, between 1700 and 2000 AD, the terrestrial biosphere transitioned from being mostly wild to mostly anthropogenic, passing the 50% threshold early in the 20th century [8]. This transformation makes it important to consider human influence on modern fire regimes [9]. 

Guyette et al. (2002) [9] identified four ways in which human influence the amount of land burnt (or the burned area fraction): anthropogenic ignitions, fuel production, fuel fragmentation and cultural behaviour. All these factors are linked to population density. Many regional studies show a single-peaked relationship between human population and fire extent and/or numbers of fires, with intermediate populations at the peak of this parabola, after which different land use activities and land cover types attenuate fire frequency and reduces burnt area fraction [10-13]. 

The objective of this study is to investigate the influence of population density on burnt area by exploring its spatial variability using Geographically Weighted Regression, and try to detect existence of critical thresholds in population density for fire behaviour using quantile regression. We then interpret the findings in the light of differences in major land use management classes.

## Data and Methods

### Data

**Figure 1:**
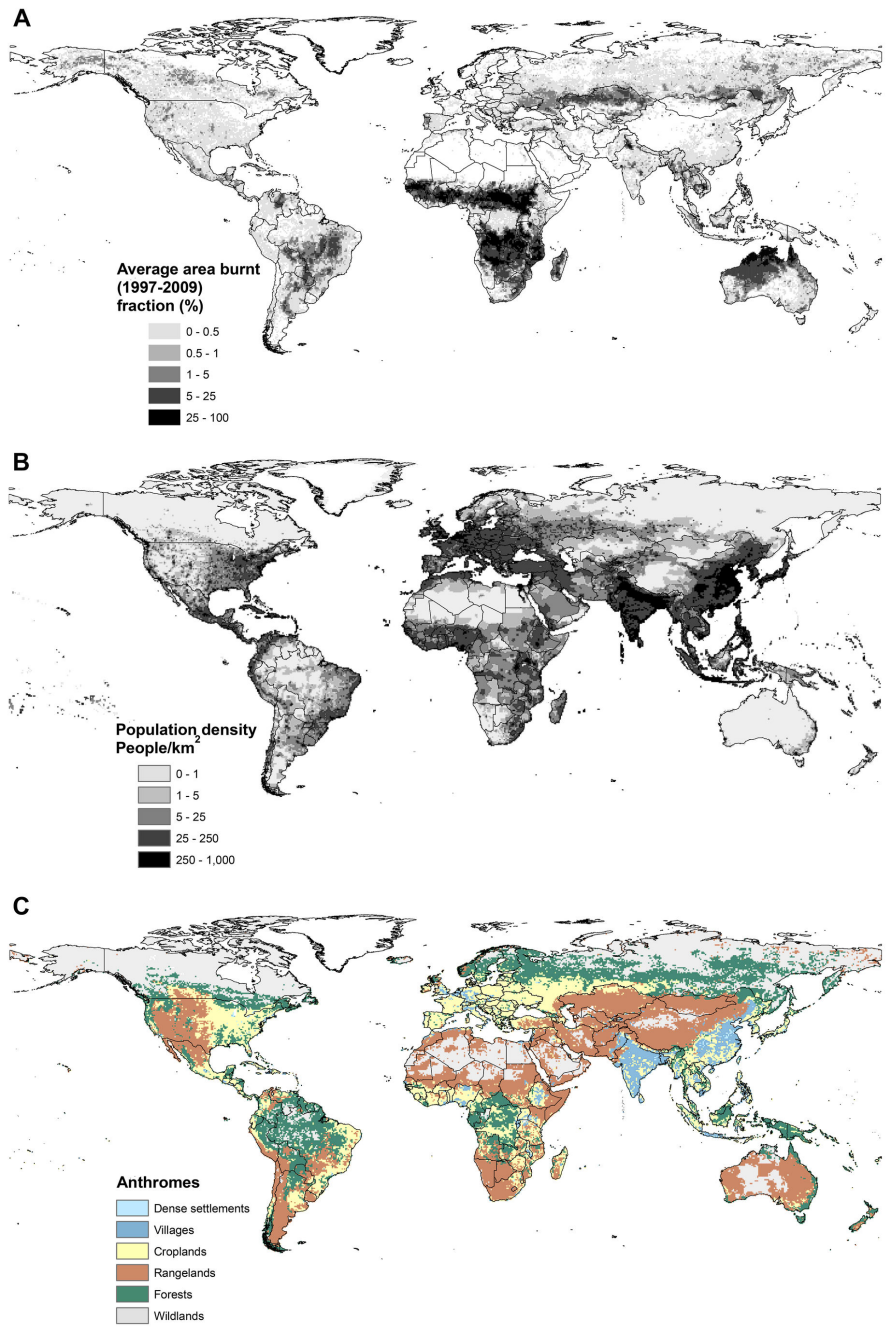
Input data sets. Average mean annual burned area (showed in cell area fraction instead of km^2^ in order to help the interpretation), based on data from the Global Fire Emissions Database version 3 (GFED3: Giglio et al., 2010) for the period 1997-2009; (B) Population density (persons per square kilometre: p/km^2^) from the Gridded Population of the World version 3 (Ciesin, 2005); and (C) The anthropogenic biomes (anthromes) of the world, mapped as the six major anthrome types (see Table 1) defined by Ellis and Ramankutty (2008).

Satellite-derived burned area maps covering 13 years (1997-2009) are available from the Global Fire Emissions Database version 3 (GFED3: [14]) at 0.5° cell resolution for the whole globe (Fig. 1a), available at: http://www.globalfiredata.org/. This spatial resolution can reveal first-order global and continental-scale patterns in burnt area [15]. Giglio et al. (2010) [14] demonstrated that the GFED v3 data used in this study has improved accuracy over version 2 in Canada and the USA. Since active fire detection can capture mush smaller events (sub-pixel) than burned area products, GFED may indeed better represent area burned in small fires than products that do not rely on active fire data. For 0.5° spatial resolution burned area, GFED v3 uses either VIRS or ATSR world fire atlas fire counts [14]. The input data for a GWR are the centroids of the 0.5° cells. Cells that intersect water bodies, ice and artificial surfaces are considered to be non-combustible areas and were removed using a mask from the Global Land Cover 2000 database [16]. The global combustible area extent was calculated from the area of each cell using a latitude correction. The annual mean burned area (km^2^) for the 13 years of observations was used as the response variable. Population density (persons per square kilometre: p/km^2^) was obtained from the Gridded Population of the World version 3 [17] at 0.5° spatial resolution (Fig. 1b) available at: http://sedac.ciesin.columbia.edu/data/collection/gpw-v3, and is used as the predictor variable. As both burned area and population density are highly skewed toward small values, we applied a decimal logarithmic transformation to both variables.

To support the interpretation of our analyses of the human influence on burned area, we use the anthropogenic biomes (Anthromes) of the world [18] available at: http://ecotope.org/anthromes/v1/guide/. This dataset classifies terrestrial biomes based on the level of human influence, estimated as a function of population density, land use and land cover. The 21 anthromes are grouped into 6 major anthrome types in the original publication (Table 1; Figure 1c) and we use these major types here to simplify interpretation of the GWR results.

**Table 1:** Anthromes and major anthrome types defined by Ellis and Ramankutty (2008).

**Anthrome**	**Major anthrome type**
Urban	Dense Settlement
Dense settlements	Dense Settlement
Rice villages	Villages
Irrigated villages	Villages
Cropped and pastoral villages	Villages
Pastoral villages	Villages
Rainfed villages	Villages
Rainfed mosaic villages	Villages
Residential irrigated cropland	Croplands
Residential rainfed mosaic cropland	Croplands
Populated irrigated cropland	Croplands
Populated rainfed cropland	Croplands
Remote croplands	Croplands
Residential rangelands	Rangelands
Populated rangelands	Rangelands
Remote rangelands	Rangelands
Populated forest	Forest
Remote forest	Forest
Wild forest	Wildlands
Sparse trees	Wildlands
Barren	Wildlands

### Statistical Analyses

We initially computed the global linear relationship between burned area and population density using Ordinary Least Squares (OLS). The global OLS regression model assumes that the studied relationship is stationary, i.e. the estimated parameters do not vary spatially. To test the hypothesis that the relationship varies spatially, we use Geographically Weighted Regression (GWR). GWR estimates local parameter values as in (Eq. 1) [19,20].

$$y=\beta_{o}(\mu ,\nu )+\sum_{j=1}^{p}\beta_{j}(\mu ,\nu )X_{j}+\varepsilon$$ (1)

Where (μ,ν) is the coordinate location and j is the number of the explanatory variables of the X matrix, β is a matrix with the regression coefficients and ε is a random error whose distribution is N(0, σ^2^I) [20].

We initially ran the GWR at 0.5° spatial resolution, which is the original resolution of both the burned area and population data sets, but also the resolution used by several Dynamic Global Vegetation Models (DGVM). Other than such pragmatic criteria, the choice of an appropriate level of spatial aggregation for analyses of spatial relationships is essentially arbitrary [21]. The basic assumption of the GWR is that observations closer to a target point have more impact on the modelled relationship at that point than more distant observations. A distance decay function centered on each observation is used for this purpose, and this makes it important to choose an appropriate level of aggregation. The GWR procedure includes a step that assesses whether the selected scale is appropriate. However, to assess the sensitivity of the spatial relationship between population density and burned area to the choice of spatial resolution, we used Africa as a test case and re-ran the analyses for this continent using 0.25° and 1.0° cells. The distance-decay depends on the bandwidth of the spatial kernel used, which is the radius or the number of observations around each point [19]. Here, we used a continental space scale, defining the continents according to political borders (M. Charlton, personal communication). We used an adaptive Gaussian kernel, whose bandwidth varies according to the density of the data, an approach usually adopted when there is no prior knowledge of the studied relationship [19]. The optimal bandwidth for each continent was determined by minimizing the Akaike information criterion (AIC) [22,23]. Due to the different extent of land of each continent and the minimization of the AIC coefficient for the highest adjusted R^2^, the bandwidth varies, but remains proportional at 5.1% of the total observations in all cases. The use of the AIC ensures that we use the appropriate level of spatial aggregation for each continent. The analysis is performed with GWR version 3.0. (see 20).

A Monte Carlo permutation test is used to test the significance of the spatial variability of local coefficient estimates. We only map the statistically significant values of the GWR output parameters (slope coefficients and intercept), as determined by a t-test. As multiple hypotheses tests are used, an alpha correction is employed to reduce type I errors [23]. The parameter coefficients were tested for significance according to the family-wise error rate ξ_m_ by choosing 

$$a=\frac{\xi_{o}}{1+p_{e}-\frac{p_{e}}{np}}$$ (2)

where p_e_ is the effective number of parameters, n is the total number of observations and p is the number of parameters in each model [23,24].

The relationship between population density and burned area is expected to be non-linear and non-monotonic. To examine whether there are abrupt changes in the nature of the relationship switches, we fit a linear ''broken stick'' version of quantile regression [25-28], using the package ''quantreg'' in R (http://cran.r-project.org/web/packages/quantreg/index.html). This technique makes no prior assumption of abruptness [29]. Since there could be more than a single slope in rate of change because of interactions between factors [30], we consider the 50^th^ and 90^th^ burned area percentiles, to explore the impact of human activities on area burnt. 

## Results

### OLS versus GWR comparison

The relationship between log-transformed population density and burned area estimated using OLS is poor, with R^2^ values varying from 0.001 (Australia) to a maximum of 0.27 (Africa). The relation between the log-transformed population density and burned area using the GWR models at 0.5° resolution is significant for all continents; the proportion of variability in burned area explained varies from 46% (Asia) to 80% (Africa). Thus, GWR performs much better than OLS for every continent. The GWR models also have lower AIC coefficients than OLS model and much higher adjusted R^2^ values (Table 2), showing that the local model (GWR) is a significant improvement on the global (OLS) model for all continents. 

**Table 2:** Percentage of statistically significant values per continent, AIC coefficients and adjusted R^2^ for the OLS and the GWR model.

**Continent**	**Number of observations**	**% significant slopes**	**% positive slopes**	**AIC (OLS)**	**AIC (GWR)**	**Adjusted R^2^ (OLS)**	**Adjusted R^2^ (GWR)**
**Africa**	10647	61.3	75.5	52871.22	39362.11	0.27	0.80
**Asia**	23799	65.7	78.3	115337.97	101721.13	0.04	0.46
**N. America**	17192	42.9	55.1	76372.24	67573.16	0.24	0.55
**S. America**	6551	46.8	81.8	31317.89	26493.67	0.07	0.56
**Europe**	6899	21.44	68	32402.14	27009.41	0.11	0.60
**Australia**	3038	20.51	76.4	14508.43	11947.93	0.001	0.58

The GWR analysis shows that there is no significant relationship between population density and burned area over 48.5% of the total area of the globe. A significant relationship between population density and burned area is found for ca 66% of the total area of Asia, ca 61% of Africa, ca 47% of South America and 43% of North America. Less than 22% of Europe and Australia are characterised by a significant relationship between burned area and population density.

### Spatial patterns

**Figure 2:**
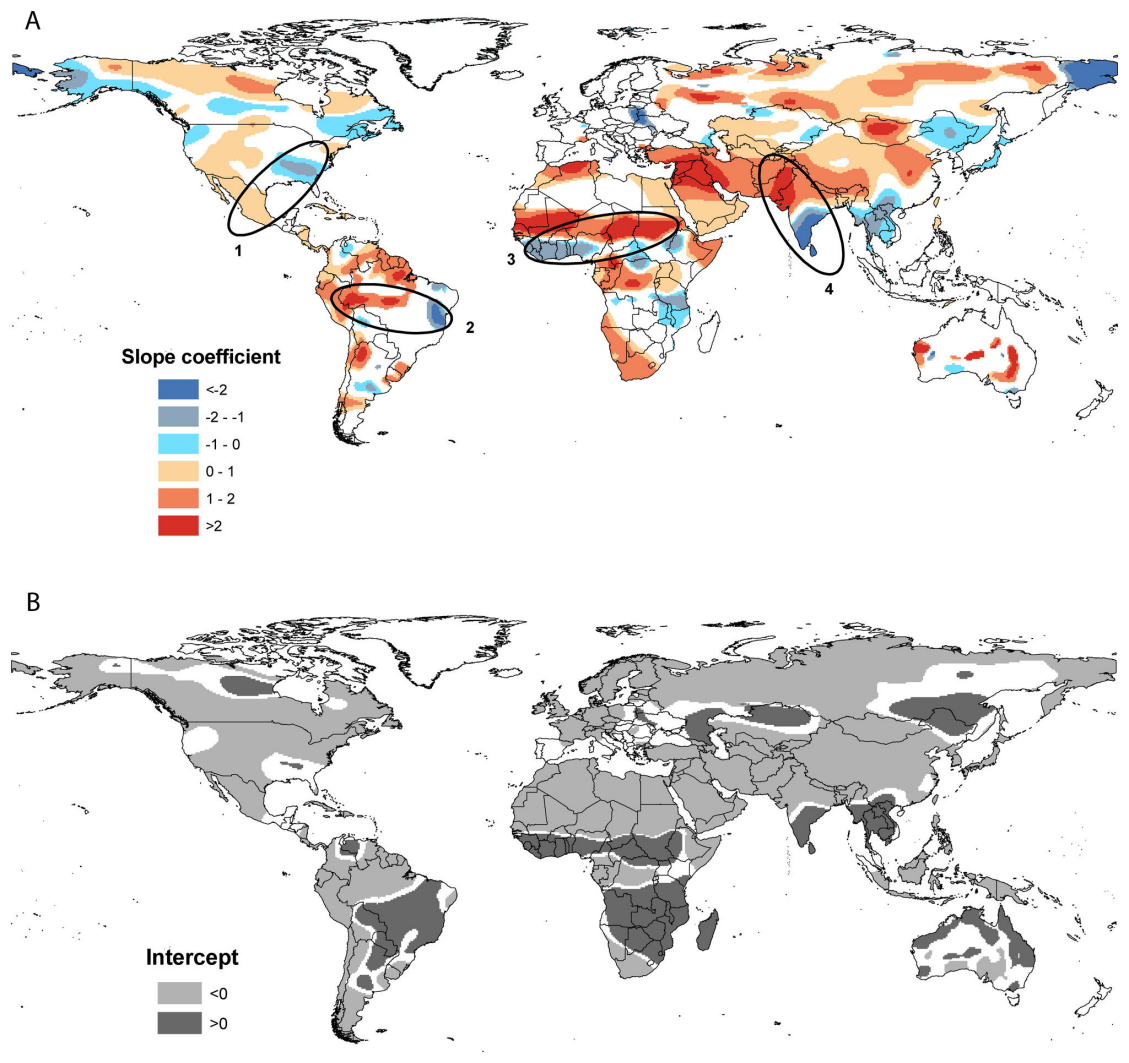
Output parameters and local R^2^. The upper panel (A) shows results from the GWR analysis, showing the nature of the relationship between population density and burned area for those regions where the relationship is significant at the 95% level (red shows a positive relationship, blue shows a negative relationship). The slope coefficient classes are defined separately for each continent; (B) Mapped patterns in the sign and magnitude of the statistically significant intercept values from the GWR analysis of population density and burned area. Dark grey shows positive intercept values, where the area burned is large even at negligible population density (i.e. where the landscape is naturally fire-prone), while light grey shows negative intercept values where climatic or vegetation factors do not favour fire; (C) Showing the significant patterns for both intercept and slopes according to the sign of the relationship, thus the four combinations show both out being positive (red), positive slopes and negative intercept (green), negative slopes and positive intercept (yellow) and both being negative (blue) ;(D) Mapped patterns of the local R^2^ for the regions where the relationship between population density and burned area is statistically significant.

The relationship between population density and burned area is non-stationary and shows patterns that differ both in sign and magnitude (Figure 2a). The relationship can be positive or negative, where positive relationships indicate that human activities increase burned area and negative relationships indicate that human activities have negative influence on fire. The magnitudes of the slope coefficients are different in different continents, so we focus on the sign in regions showing significant relationships between population density and burned area. The intercept can be an estimate of the area burnt when population density is small (1 p/km^2^), although absolute values are influenced by the slope coefficient. However, for regions displaying similar slopes, the intercept can be interpreted as a measure of the fire-proneness of the landscape, where positive intercepts indicate that the region is fire-prone and negative intercepts indicate that the region is less likely to burn. 

### Africa

On average over the period 1997-2009, 69% of the global area burned is in Africa. This is comparable to Tansey et al.’s (2004) [31] estimate that Africa accounted for 64% of the total area burned in 2000. The relationship between population density and burned area is statistically significant over nearly 61% of the continental area, with positive relationships in the Maghreb, the Sahel, the Horn of Africa, central Africa, and south-western Africa (Figure 2a) and negative relationships in the Sudanian savannah region and parts of eastern Africa (Figure 2a). Over most of these areas, the slope coefficients are >1 (or < -1), indicating that the impact of people becomes progressively larger at higher population densities. Regions with positive relationships between burned area and population density generally occur in rangelands (Figure 1c), while the areas with negative relationships have a higher incidence in areas of croplands and villages, and in forests. Absence of fire (because of lack of fuel) explains the absence of significant relationships in the Sahara, but the absence of any significant relationship associated with the high burned area (Figure 1a) in the Angola-Congo-Zambia region is perhaps more surprising (Figure 2a), given the general view that Miombo woodlands are highly susceptible to anthropogenic fires (e.g. 32) and has been identified as one of the highest fire incidence in the world [15]. Population density in this region is rather low (5-15 people per km^2^) and shows little spatial variability. This could partly explain the lack of a significant relationship between burned area and population. However, the region is characterised by a mosaic of open, fire-prone savannah vegetation and it is likely that both the high incidence and variability of fire is mainly determined by variability in climate and vegetation.

About 40.1% of the regions showing a significant relationship between burned area and population density have positive values for the intercept (Figure 2b), most particularly the Sudanian savannah region and parts of eastern Africa, where the relationship between fire and population density is inverse (Figure 2a). The positive intercept values indicate that these regions are naturally fire-prone, and help to explain why increasing population density should lead to lower area burnt. In contrast, the regions with negative intercepts can be interpreted suggesting that low fuel loads would normally limit fire and human modification of the vegetation cover is responsible for the relatively high levels of fire in these regions. This is consistent with the finding that burned area increases strongly (values > 1) with population density, because landscape modification will also increase with population density.

### Asia

Asia is the continent with the largest area (over 49 million km^2^) and the highest percentage (66%) of statistically significant slope values (Table 2). There are three regions that show a positive effect of population. The first extends from Turkey and Saudi Arabia through Iran and across to Afghanistan, Pakistan and northern India, the second is the rangelands of Mongolia and northern China, and the third occurs in the boreal parklands of Russia. Whereas the slope coefficients of the first two regions are always >1 (Figure 2a), a large part of the Russian parklands has slope coefficients <1, showing that the largest effects on fire occur for small population increments. Regions with significant negative slope coefficients occur in southern India, Southeast Asia and southern China, north-eastern China and Chukotka (north-eastern Siberia). As in the case of Africa, the regions showing positive relationships between burned area and population density tend to be predominantly characterised by rangelands. Regions characterised by villages and croplands, rangelands with extremely high population densities (e.g. in northern India and Pakistan, where populations densities are >250 p/km^2^) and forested areas tend to show positive relationships between burned area and population density.

About 14% of the area of the total significant intercept values are positive (Figure 2b). Positive intercepts occur in southern India, Southeast Asia and north-eastern China – all areas where the relationship between burned area and population density is negative (i.e. an increase in population leads to suppression of fire). Areas with negative intercept values in the region stretching from Turkey through northern India to Mongolia show positive relationships between burned area and population density. The relationship in the boreal parklands is more complex, since although areas with positive slope coefficients mostly have negative intercept values, there are some limited areas with positive slope coefficients. However, the R^2^ values (Figure 2d) in these regions are low (< 0.25), and the difference in the signals may not be robust. A different relationship occurs in Chukotka (and indeed in parts of Alaska), where negative relationships between burned area and population density are characterised by negative intercepts. Thus, in this not particularly fire-prone tundra region, increasing population density can significantly reduce fire incidence.

### North America

The relationship between population density and burned area is statistically significant for over 43% of the area of North America (Table 2a). Positive relationships are found in the semi-arid (and mostly rangeland areas) of northern Mexico and the Great Basin, and in the boreal parkland regions of Canada and north-eastern Alaska. Negative relationships are found in the forested and cropped landscapes of south-eastern U.S.A (Alabama, Georgia, South Carolina), the forested regions of the Pacific Northwest and southern Alaska, and the boreal forest zone of central and eastern Canada (Figure 2a). Across virtually all of North America, slope coefficients are between 1 and -1, a feature that is consistent with the high technological levels of agriculture and contrasts strongly with regions of more traditional agricultural practices, such as Africa.

Only 4.1% of the regions showing statistically significant intercept have positive values. Positive intercept values (Figure 2b) are found in the south-eastern U.S.A., where the relationship between burned area and population density is negative, and in the boreal parklands of northern Canada where the slope of the relationship is positive. Areas with negative intercepts are characteristic of the southern boreal forest in eastern Canada and southern Alaska, and the semi-arid rangelands of the Great Basin and northern Mexico. The relationship between slope and intercept in the rangeland areas (positive slope coefficients, negative intercepts) is consistent with what is observed in semi-arid rangelands in other parts of the world; the relationship in the southern boreal forests (negative slopes, negative intercepts) is distinctive.

### South America

The relationship between population density and burned area is statistically significant for 47% of the area of South America (Table 2). The relationship is positive around the margins of Amazonia, and in northern Argentina (Figure 2a). Negative relationships are found in the Bahia state in Brazil and, somewhat anomalously, in the rangeland area of central Argentina. The areas characterised by negative relationships between burned area and population density have positive intercepts (Figure 2b), i.e. these are fire-prone areas where increasing population leads to a reduction in fire. Most of the regions where the relationship between burned area and population density is positive are characterised by negative intercept values. However, in some parts of the so-called “arc of deforestation” on the southern side of the Amazon forest the positive relationship between fire and population density is associated with positive intercept values (Figure 2b), indicating fire-prone landscapes where human activity is increasing the amount of burning.

### Europe and Australia

The relationship between population density and area burned is significant in < 22% of Europe and Australia (Table 2). In Europe, the only areas showing statistically significant negative relationships are in Poland, Ukraine and Belarus. However, intercept values in this region are positive, indicating some level of landscape susceptibility to fire. This feature most likely reflects a pattern dominated by the forested landscapes of e.g. the Carpathians, where natural forest fire regimes are suppressed with increasing population. Positive relationships between population density and burned area are found in some forested parts of north-western Russia; the intercept values are negative.

There are no significant relationships between area burned and population density in the savannah and rangeland areas of northern and western Australia characterised by the highest incidence of fire in the continent (Figure 1a). Areas showing a positive relationship between burned area and population density are in the rangelands of the Murray-Darling basin, on the northern margin of the Lake Eyre basin and to the south of the Hamersley Range in Western Australia. These regions are all associated with positive intercept values. Small areas showing a negative relationship between burned area and population density occur on rangelands of the Nullarbor Plain and in the densely-settled region (Figure 2a) around Melbourne in southern Victoria. The Nullarbor is characterised by negative intercept values (Figure 2b), presumably because of the very low vegetation cover and hence fuel loads in this region. In contrast, the area around Melbourne is characterised by positive intercept values – this is a fire-prone area where human activities work to suppress fire. 

### Sensitivity to spatial resolution

As expected, the GWR is sensitive to the choice of spatial resolution (Table 3). The extent of the area with significant relationships decreases with spatial resolution, from 68% at 0.25° resolution to 52% at 1° resolution. Changing resolution does not affect the conclusion that most of the relationships between burned area and population density are positive, but the proportion of positive values increases with increasing resolution, reaching an unrealistically high value of 98% at 1° resolution. This presumably reflects the increasing homogenisation of grid cell values of burnt area and/or population density with increasing resolution. The geographical patterns of significant values, and positive and negative relationships, are not impacted by the change from 0.25° resolution to 0.5° resolution, although areas showing negative relationships between population density and burned area have virtually disappeared in the analyses at 1° resolution. These sensitivity tests suggest that the overall conclusions of our GWR analysis would not be affected by running at finer than 0.5° resolution, but clearly information is likely to be lost in analyses at coarser resolution.

**Table 3:** Summary statistics of the GWR analysis for Africa, comparing the results obtained using 0.25°, 0.5° and 1.0° grid cell resolutions.

**Continent**	**Number of observations**	**% significant slopes**	**% positiveslopes**	**AIC (OLS)**	**AIC (GWR)**	**Adjusted R^2^ (OLS)**	**Adjusted R^2^ (GWR)**
**Africa (0.25)**	40864	67.7	65.5	189463.45	138841.26	0.21	0.77
**Africa (0.5)**	10647	61.3	75.5	52871.22	39362.11	0.27	0.80
**Africa (1)**	2557	52.4	97.8	12261.02	8188.66	0.36	0.87

### Impact of land-use on the relationship between burned area and population density

**Figure 3:**
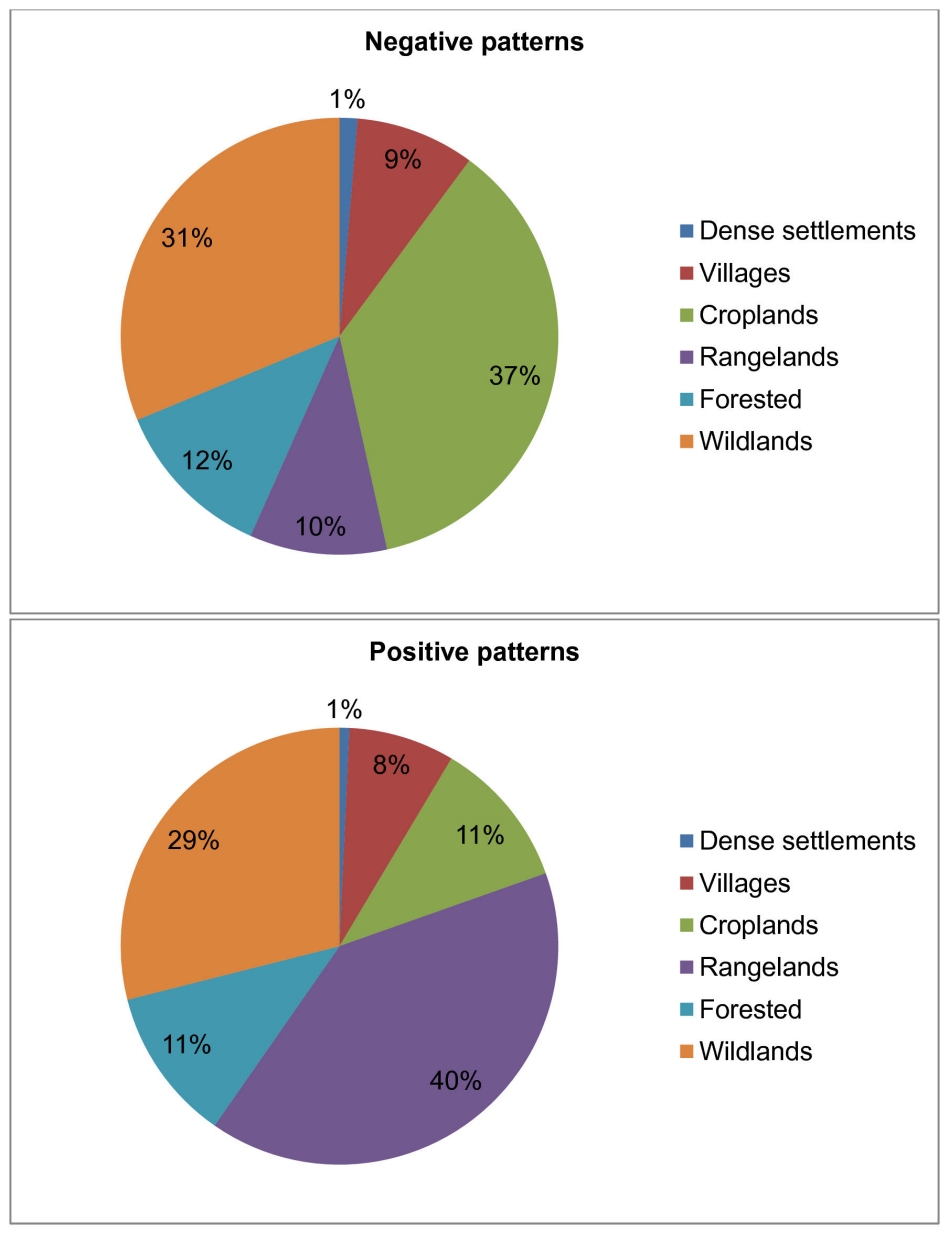
Proportion of Anthromes showing negative and positive relationship. Pie-charts showing the relative proportion of the total area showing (A) positive and (B) negative significant relationships between burned area and population density, classified according to the six major anthrome types defined by Ellis and Ramankutty (2008).

Both positive and negative relationships between burned area and population density are found in every type of anthrome (Figure 3). Wild lands represent a significant proportion of the regions where there is a significant correlation between burned area and population density, but nevertheless the proportion showing positive or negative correlations is about the same (29% of the total area showing positive, 31% showing negative correlations). However, croplands and rangelands are not equally represented in the two classes of relationship: 37% of the area where there is a negative relationship between burned area and population density is cropland and only 10% is rangelands. Conversely, rangelands account for nearly 40% of the area where there is a positive relationship between burned area and population density, while croplands account for only 11% of these regions. 

### Quantile regression analysis

Although regions may show an overall positive or negative relationship between burned area and population density, the nature and strength of the relationship is not necessarily constant at different levels of population [33]. We examine whether there are critical thresholds in population density at which the relationship between population density and the burned area extent changes using quantile regression, focusing on four different regions in Africa, Asia and the Americas (Figure 2a). Each region is characterised by close bipolar patterns, thus displaying areas with both strongly positive and strongly negative slope coefficients (Figure 2a). 

**Figure 4:**
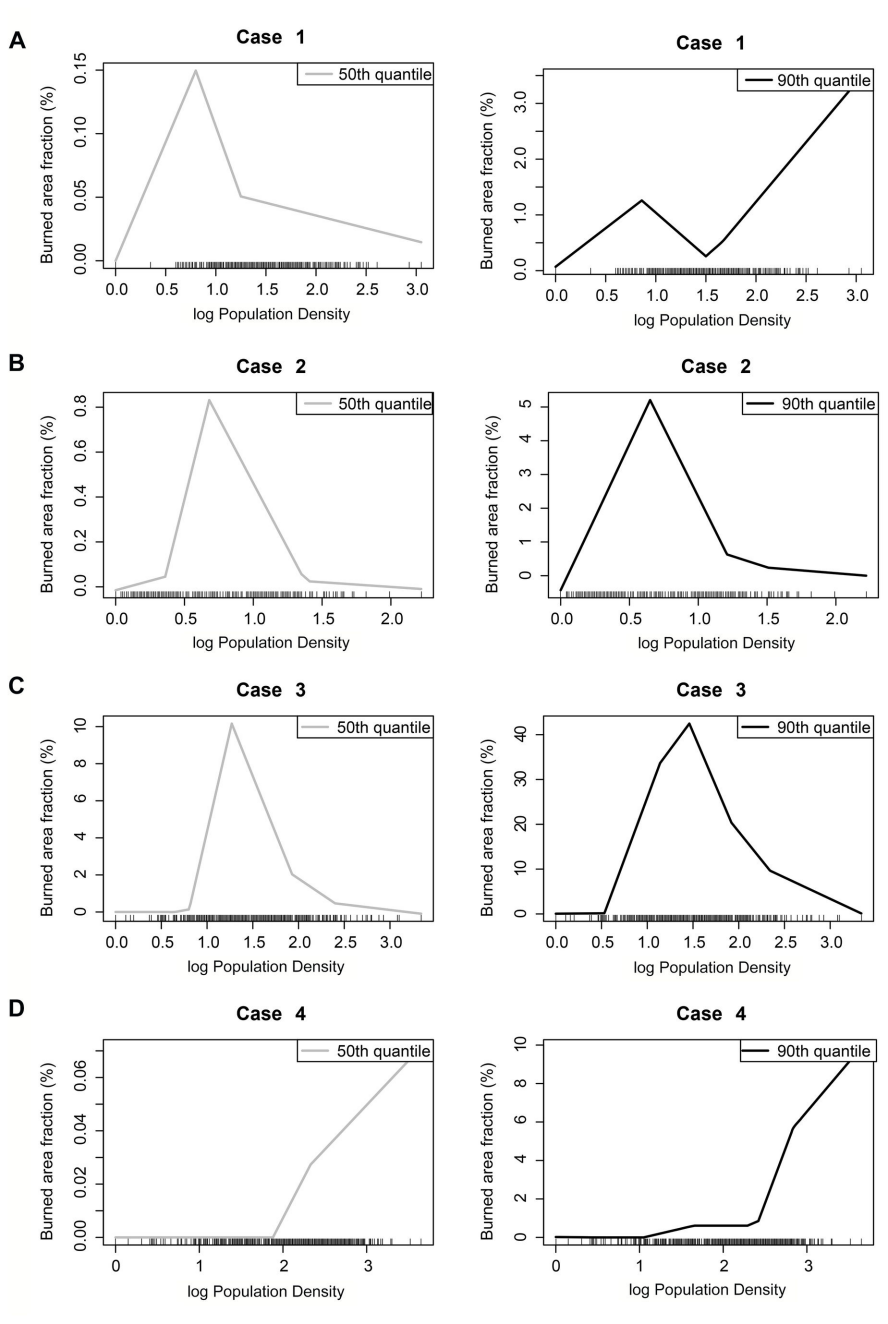
The relationship between burned area and population density at different levels of burned area (50 and 90%) for the 4 case studies. (A) Case 1 in North America. (B) Case 2 in South America. (C) Case 3 in Africa. (D) Case 4 in Asia.

In Asia, the relationship between population density and burned area is monotonic (Figure 4, case 4): as population density increases the impact on fire, whether positive or negative, increases. This is true for regions with moderate levels (50%) and at higher levels of burned area. In Africa and South America, the relationship between population density and burned area is non-monotonic: burnt area increases up to a given threshold, reaches a peak and then declines (Figure 4, cases 2-3). In both regions, the change point in the nature of the relationship occurs at about 7 p/km^2^ in both regions with moderate and high levels of fire. The impact of changes in population density on burned area becomes negligible at population densities greater than ca 10 p/km^2^ in both regions. The relationship between burned area and population density in North America is also non-monotonic (Figure 4, case 1). In regions with only moderate levels of fire (as shown by the 50% quantile regression), the relationship is similar to that observed in South America and Africa: increasing population density leads to increasing impact on burned area up population densities of 7 p/km^2^ and then becomes negative and the slope becomes more gentle at population densities of > 12 p/km^2^. However, in regions with higher levels of fire (as shown by the 90% regressions), the negative relationship at population densities > 7 p/km^2^ is reversed and becomes positive at population levels > 30 p/km^2^. 

## Discussion and Conclusions

This study quantifies the spatial variability in the relationship between human activities (as measure by population density) and fire (as measured by burned area). There is no statistically significant relationship between burned area and population density over more than 50% of the global land area. The univariate relationship between population density and area burnt is relatively unimportant in Australia and Europe: in the case of Australia this supports the idea of a strong climate control on fire regimes, while in Europe the lack of relationship most likely reflects the closely managed nature of the landscape. At a sub-continental scale, there are regions where population density has little or no impact on burned area. In Kazakhstan, for example, variability in burning shows no relationship with population density despite the fact that this is one of the biggest agricultural areas in the world [34] and the country that contributes with highest amount of area burnt in dry land ecosystems in central Asia [35]. A similar situation pertains in the Miombo woodlands region of Angola-Congo-Zambia, where variability in burned area is unrelated to population density. The absence of a relationship between population density and burned area over much of the globe does not imply that human activities have no influence on fire regimes. Several studies have shown, for example, that humans can alter the timing [36] or the number of fires [13,37]. However, since it is burned area (rather than timing or number) that is most important for the carbon cycle and pyrogenic emissions, the lack of a strong relationship between human activities and burned area over much of the globe, including areas characterised by high levels of burning, is noteworthy.

In areas where there is a statistically significant relationship, this relationship is positive (i.e. burned area increases with population density) over 73.3% of the global land area. However, there are substantial parts of the world, where the relationship between burned area and population density is negative (i.e. increasingly human activity leads to fire attenuation). The relative proportion of the land area showing positive/negative relationships varies from continent to continent. There has been considerable focus on the positive relationship between human activities and burned area, through using fire to clear land and as part of the agricultural regime [34,38]. The impact of landscape fragmentation on reducing fire in agricultural area has also been documented [16,39,40]. 

About 30% of the regions where a significant relationship between population density and burned area are classified as wild lands, but the relative proportion of wild lands in the areas showing positive or negative relationships between fire and human activity is approximately the same. This is not the case for all anthromes. Rangelands are over-represented in the regions showing positive relationships between fire and population, while croplands are over-represented in the regions showing negative relationships between fire and population. The distribution of rangelands is to some extent a reflection of climate controls, with most rangeland areas occurring in semi-arid regions and croplands occurring in more well-watered regions, nevertheless these tendencies suggest that land-use practices can have a significant impact of fire regimes. 

The GWR analysis shows that, in general, regions which display a negative relationship between burned area and population density generally have positive intercept values, and vice versa. In other words, in regions where climate and/or vegetation create conditions where fires are likely (i.e. fire prone landscapes), people tend to supress fire whereas in regions that are less fire-prone because of e.g. lack of fuel, people tend to increase the area burned. However, there are regions where there is both a positive relationship between population and burned area and the intercept is also positive. One of these regions is the arc of deforestation on the southern border of Amazonia, suggesting that deforestation is exploiting a landscape that is already susceptible to the impact of fire. This is not a new suggestion; Le Page et al. (2010) [41] pointed out that this region experiences three to five months of low precipitation which facilitates extended periods of burning. 

In most regions of the world, the nature of the relationship between population density and burned area is non-monotonic: increasing human activity (as measured by population density) initially lead to an increase in burned area but this peaks at intermediate levels of population density and then declines. The critical value in three of the case studies regions examine here is around 7 p/km^2^ and above values of 12 p/km^2^ there is no further impact of population density changes on fire. Our results for Africa support previous findings [13,42] that suggest that fire extent in this region displays a non-monotonic relationship with anthropogenic variables. However, in areas of North America characterised by high fire, there is a second threshold at ca > 30 p/km^2^ where increasing population density leads to increasing burned area. The situation in Asia seems anomalous in that increasing population density always leads to an increased impact in burned area.

The GWR approach allows an appropriate spatial scale of comparison to be selected continent by continent through the choice of bandwidth combined with use of the AIC. Fotheringham et al. (2002) [20] showed that the GWR approach was more robust to the choice of spatial resolution than models that do not take spatial non-stationarity into account. Nevertheless, we examined the impact of the choice of spatial resolution using Africa as a test case. The use of a higher resolution than our baseline of 0.5° produces no change in the geographic patterns of regions showing significant positive or negative relationships between population density and burnt area, although the percentage of grid cells showing positive values declines slightly (and the percentage showing negative values correspondingly increases). The overall impact of increasing the resolution is slight, and this suggests that our regional findings are robust. Most other studies of the controls on burnt area have used coarser spatial resolutions (e.g. 12,43). Our sensitivity analyses show that decreasing the resolution has a larger impact on the geographic patterns, and particularly on the recognition of areas where the relationship between population density and burnt area is negative. This suggests that these earlier studies may miss important aspects of the relationship between population density and burnt area because of their choice of spatial scale. The selection of spatial scale is can affect the conclusions about the nature of spatial relationships, making it important to use a technique (such as the AIC optimization) that allows this choice to be made objectively.

Understanding the complexity of the relationships between people and fire is important in a modelling context. Fire-enabled dynamic vegetation models can be used to predict the consequences of projected changes in climate on fire regimes (see e.g. 44-46). However, those models that explicitly include anthropogenic fire generally focus on human impacts on ignitions and furthermore employ either a universal population density value as a threshold for anthropogenic fire ignitions [47], or single-peaked global function of population density [48]. Other fire-enabled DGVMs (e.g. LPX: [49]) ignore anthropogenic ignitions, although they allow for human suppression of fire in agricultural areas. No extant model incorporates spatially varying relationships between burned area and population density that are dependent on vegetation types, land-use and cultural practices – which our analyses show a non-negligible influence on regional fire regimes. However, this study has not exhausted the analyses necessary to arrive at a complete understanding of the biogeography of fire.

## References

[1] Bond WJ, Wilgen B (1996) Fire and plants. Chapman and Hall London. UK.

[2] Johnson E (1996) Fire and Vegetation Dynamics: Studies from the North American Boreal Forest. Cambridge University Press Cambridge.

[3] Pyne SJ, Andrews PL, Laven RD (1996) Introduction to wildland fire. New York, John Wiley and Sons p.769.

[4] Pyne SJ (2009) The human geography of fire: a research agenda. Progress in Human Geography. 33(4): 443–446. doi:10.1177/0309132508101598.

[5] Flannigan MD, Stocks BJ, Wotton BM (2000) Climate Change and Forest Fires. 1–9. Sci Total Environ 262(3): 221-229. doi:10.1016/S0048-9697(00)00524-6. PubMed: 11087028. 11087028

[6] IPCC (2007) Climate Change 2007: The Physical Science Basis. Contribution of Working Group I to the Fourth Assessment Report of the Intergovernmental Panel on Climate Change [ Solomon S Qin D Manning M Chen Z Marquis M Cambridge University Press, Cambridge, UK and New York, NY, USA.

[7] Sanderson EW, Jaiteh M, Levy MA, Redford KH, Wannebo AV et al. (2002) The Human Footprint and the Last of the Wild. BioScience, 52(10): 891-904.

[8] Ellis EC, Goldewijk K, Siebert S, Lightman D, Ramankutty N (2010) Anthropogenic transformation of the biomes, 1700 to 2000. Global Ecology and Biogeography 19(5). doi:10.1111/j.1466-8238.2010.00540.x.

[9] Guyette RP, Muzika RM, Dey DC (2002) Dynamics of an anthropogenic fire regime. Ecosystems 5: 472-486. doi:10.1007/s10021-002-0115-7.

[10] Barbosa PM, Stroppiana D, Gregoire J-M, Pereira JMC (1999a) An assessment of vegetation fire in Africa (1981–1991): burned areas, burned biomass, and atmospheric emissions. Glob Biogeochem Cycles 13(4): 933–950. doi:10.1029/1999GB900042.

[11] Syphard AD, Radeloff VC, Keeley JE, Hawbaker TJ, Clayton MK et al. (2007) Human influence on California fire regimes. Ecol Appl 17(5): 1388–1402. doi:10.1890/06-1128.1. PubMed: 17708216. 17708216

[12] Aldersley A, Murray SJ, Cornell SE (2011) Global and regional analysis of climate and human drivers of wildfire. Sci Total Environ 409(18): 3472–3481. doi:10.1016/j.scitotenv.2011.05.032. PubMed: 21689843. 21689843

[13] Archibald S, Roy DP, vanWilgen BW, Sholes RJ (2009) What limits fire? An examination of drivers of burnt area in Southern Africa. Glob Change Biol 15(3): 613–630. doi:10.1111/j.1365-2486.2008.01754.x.

[14] Giglio L, Randerson JT, van der Werf GR, Kahsibhatla PS, Collatz GJ et al. (2010) Assessing variability and long term trends in burned area by merging multiple satellite fire products. Biogeosciences 7: 1171–1186. doi:10.5194/bg-7-1171-2010.

[15] Oom D, Pereira JMC (2013) Exploratory spatial data analysis of global MODIS active fire data. International Journal of Applied Earth Observation and Geoinformation 21: 326–340. doi:10.1016/j.jag.2012.07.018.

[16] Bartholomè E, Belward AS (2005) A new approach to global land cover mapping from Earth observation data. International Journal of Remote Sensing 26(9): 1959–1977. doi:10.1080/01431160412331291297.

[17] Center for International Earth Science Information Network (CIESIN), Columbia University, Centro Internacional de Agricultura Tropical (CIAT) (2005) Gridded population of the World version 3 (GPWv3): population density grids. Socioeconomic Data and Applications Center (SEDAC), Columbia University, Palisades.

[18] Ellis EC, Ramankutty N (2008) Putting people in the map: anthropogenic biomes of the world. Frontiers in Ecology and the Environment 6(8): 439–447. doi:10.1890/070062.

[19] Brunsdon CA, Fotheringham AS, Charlton ME (1998) Geographically weighted regression–modelling spatial non-stationarity. Stat 47(3): 431–443.

[20] Fotheringham S, Brunsdon C, Charlton M (2002) Geographically Weighted Regression: the analysis of spatially varying relationships. John Wiley & Sons, UK.

[21] Openshaw S, Taylor PJ (1981) The modifiable areal unit problem. in: Quantitative geography: a British View, (eds) N. Wrigley and R.J. Bennett, (Routledge and Kegan Paul: London), p. 60-70

[22] Akaike H (1981) Likelihood of a model and information criteria. J Econ 16(1): 3–14. doi:10.1016/0304-4076(81)90071-3.

[23] Byrne G, Charlton M, Fotheringham S (2009) Multiple dependent hypothesis tests in geographically weighted regression. In: Lees BG Laffan SW 10th International conference on geocomputation. UNSW, Sydney November-December

[24] Brunsdon C, Charlton M (2011) An assessment of the effectiveness of multiple hypothesis testing for geographical anomaly detection. Environment and Planning B: Planning and Design 38(2): 216 - 230. doi:10.1068/b36093.

[25] Toms JD, Lesperance ML (2003) Piecewise Regression: A tool for identifying ecological thresholds. Ecology 84(8): 2034-2041. doi:10.1890/02-0472.

[26] Koenker R, Basset G (1978) Regression Quantiles. Econometrica 46(1): 33-50. doi:10.2307/1913643.

[27] Koenker R (2005) Quantile Regression. Cambridge University Press Cambridge. UK.

[28] Sankaran M, Hanan NP, Scholes RJ, Ratnam J, Augustine DJ et al. (2005) Determinants of woody cover in African savannas. Nature 438(7069): 846–849. doi:10.1038/nature04070. PubMed: 16341012. 16341012

[29] Chiu GS (2003) Bent cable Regression for Assesing Abruptness of Change. PhD Thesis, University of British Columbia: Canada.

[30] Cade SB, Noon BR (2003) A gentle introduction to quantile regression for ecologists. Front Ecol Environment 1(8): 412-420. Available online at: doi:10.1890/1540-9295(2003)001[0412:AGITQR]2.0.CO;2

[31] Tansey K, Gregoire J-M, Stroppiana D, Sousa A, Silva J, et al. (2004) Vegetation burning in the year 2000: global burned area estimates from SPOT VEGETATION data. J Geophys Res 109: D14S03 DOI 10.1029/2003JD003598.

[32] Ryan CM, Williams M (2011) How does fire intensity and frequency affect miombo woodland tree populations and biomass? Ecol Appl 21: 48-60. doi:10.1890/09-1489.1. PubMed: 21516887. 21516887

[33] Archibald S, Staver AC, Levin SA (2012) Evolution of human-driven fire regimes in Africa. Proc Natl Acad Sci U S A 109(3): 847-852. doi:10.1073/pnas.1118648109. PubMed: 22184249. 22184249 PMC3271907

[34] Leff B, Ramankutty N, Folley JA (2004) Geographic distribution of major crops across the world. Global Biogeochemical Cycles 18.

[35] Loboda TV, Giglio L, Boschetti L, Justice CO (2012) Regional fire monitoring characterization using global NASA MODIS fire products in dry lands of Central Asia. Front. Earth Sci. 6(2): 196-205. doi:10.1007/s11707-012-0313-3.

[36] Page Le Y, Oom D, Silva JMN, Jonsson P, Pereira JMC (2009) Seasonality of vegetation fires as modified by human action: Observing the deviation from eco-climatic fire regimes. Global Ecology and Biogeography 19: 575-588.

[37] Gralewicz NJ, Nelson TA, Wulder MA (2011) Spatial and temporal patterns of wildfire ignitions in Canada from 1980 to 2006. International Journal of Wildland Fire 21(3): 230-242.

[38] Silva JMN, Carreiras JMB, Rosa I, Pereira JMC (2011) Greenhouse gas emissions from shifting cultivation in the tropics, including uncertainty and sensitivity analysis. Journal of Geophysical Research, 116 (D20) DOI 10.1029/2011JD016056.

[39] Laris P (2002) Burning the seasonal mosaic: Preventive burning strategies in the wooded savanna of southern Mali. Human Ecology 30: 155-186. doi:10.1023/A:1015685529180.

[40] Marlon JR, Bartlein PJ, Carcaillet C, Gavin DG, Harrison SP et al. (2008) Climate and human influences on global biomass burning over the past two millennia. Nature Geoscience, 1(10): 697–702. doi:10.1038/ngeo313.

[41] Page Le Y, van der Werf GR, Morton DC, Pereira JMC (2010) Modelling fire-driven deforestation potential in Amazonia under current and projected climate conditions. Journal of Geophysical Research 115: 11

[42] Sá ACL, Pereira JMC, Charlton ME, Mota B, Barbosa PM et al. (2010) The pyrogeography of sub-Saharan Africa: a study of the spatial non-stationarity of fire–environment relationships using GWR. Journal of Geographical Systems 13(3): 227–248. doi:10.1007/s10109-010-0123-7.

[43] Krawchuk MA, Moritz MA, Parisien M-A, Van Dorn J, Hayhoe K (2009) Global Pyrogeography: the Current and Future Distribution of Wildfire. PLOS ONE 4(4): e5102. doi:10.1371/journal.pone.0005102. PubMed: 19352494. 19352494 PMC2662419

[44] Scholze M, Knorr W, Arnell NW, Prentice IC (2006) A climate change risk analysis for world ecosystems. Proc of the Natl Acad of Sciences of the USA 103: 13116–13120. doi:10.1073/pnas.0601816103. PubMed: 16924112. PMC1559762 16924112

[45] Lenihan J, Bachelet D, Neilson R, Drapek R (2008) Response of vegetation distribution, ecosystem productivity, and fire to climate change scenarios for California. Clim Change 87: 215–230. doi:10.1007/s10584-007-9362-0.

[46] Harrison SP, Marlon J, Bartlein PJ (2010) Fire in the Earth System. Changing Climates, Earth Systems and Society. International Year of Planet Earth 2010, Climate Change Theme, 21-48.

[47] Venevsky S, Thonicke K, Sitch S, Cramer W (2002) Simulating fire regimes in human-dominated ecosystems: Iberian Peninsula case study. Global Change Biology 8(10): 984–998. doi:10.1046/j.1365-2486.2002.00528.x.

[48] Thonicke K, Spessa A, Prentice IC, Harrison SP, Dong L et al. (2010) The influence of vegetation, fire spread and fire behavior on biomass burning and trace gas emissions: results from a process-based model. Biogeosciences 7(6): 1991–2011. doi:10.5194/bg-7-1991-2010.

[49] Prentice IC, Kelley DI, Foster PN, Friendlingstein P, Harrison SP et al. (2011) Modeling fire and the terrestrial carbon balance. Global Biogeochemical Cycles 25. doi:10.1029/2010GB003906.

